# Downregulated Ferroptosis-Related Gene STEAP3 as a Novel Diagnostic and Prognostic Target for Hepatocellular Carcinoma and Its Roles in Immune Regulation

**DOI:** 10.3389/fcell.2021.743046

**Published:** 2021-11-01

**Authors:** Yuanliang Yan, Qiuju Liang, Zhijie Xu, Jinzhou Huang, Xi Chen, Yuan Cai, Bi Peng, Qiaoli Yi

**Affiliations:** ^1^Department of Pharmacy, Xiangya Hospital, Central South University, Changsha, China; ^2^Department of Pathology, Xiangya Hospital, Central South University, Changsha, China; ^3^Department of Oncology, Mayo Clinic, Rochester, MN, United States; ^4^National Clinical Research Center for Geriatric Disorders, Xiangya Hospital, Central South University, Changsha, China

**Keywords:** liver hepatocellular carcinoma, ferroptosis, STEAP3, prognosis, immune infiltration

## Abstract

Ferroptosis, a distinct type of regulated cell death, has been reported to be involved in the tumorigenesis of liver hepatocellular carcinoma (LIHC). However, the precise functions and potential mechanisms of ferroptosis in LIHC were still poorly understood. Herein, we investigated the biological roles of ferroptosis-related gene STEAP3 in LIHC. STEAP3 was previously proved to serve a key regulator in ferroptosis *via* mediating the iron metabolism. Comprehensive bioinformatics from several databases revealed that STEAP3 was significantly downregulated in LIHC tissues and exhibited the favorable prognostic significance in LIHC patients. The downregulated STEAP3 was further confirmed in two LIHC cells Huh7 and MHCC97H using real-time PCR and western blot. And STEAP3 overexpression significantly inhibited the cell proliferation in Huh7 and MHCC97H cells. In addition, clinical data identified the relationship between STEAP3 expression and several clinicopathological parameters of LIHC patients, including histologic grade, alpha fetal protein (AFP) concentration, etc. Receiver operation characteristic (ROC) curve revealed STEAP3 as a potential diagnostic biomarker for LIHC patients. Moreover, the co-expression network of STEAP3 was explored to gain a better insight into its underlying signaling pathways. Finally, aberrant STEAP3 might participate in varieties of immune-associated signatures in LIHC pathogenesis, including immunostimulators, immunoinhibitors, chemokines, and chemokine receptors. Taken together, these findings could enhance our knowledge regarding the inhibitory roles and underlying biological significance of STEAP3 in LIHC tumorigenesis.

## Introduction

Liver hepatocellular carcinoma (LIHC), claiming to be more than 90% among the whole cases of primary hepatoma, ranks third across all cancer-associated mortality and continues to increase rapidly in occurrence (Zhang J. et al., 2020). The prognosis of LIHC is dependent upon the stage when the neoplasm is detected and early detection of the tumor facilitates to choose ideal treatment options (Feng et al., 2020; Zhang R. et al., 2020). Unfortunately, it remains challenging to prevent advanced LIHC, and there are no survival benefits but toxic side effects when treated with systemic chemotherapy (Finn et al., 2018; Personeni et al., 2019). Recently, immunotherapy has evolved as a new standard strategy in the treatment of advanced LIHC (Ou et al., 2020). For instance, Atezolizumab combined with bevacizumab has been recommended by American Society of Clinical Oncology (ASCO) Guideline as a novel first-line therapy for most advanced LIHC patients (Gordan et al., 2020). Nevertheless, only a subset of all patients responds to immunotherapy and response rates remain limited (Ocker, 2020). Consequently, exploring novel predictive biomarkers is of relevance to improve the prognosis of advanced LIHC patients.

Ferroptosis is a distinct type of regulated cell death (RCD) linked with iron metabolism, which generally occurs accompanied by the existence of iron accumulation and lipid peroxidation (Tang et al., 2020; Wei et al., 2020). Ferroptosis could contribute to the maintenance of homeostasis as well as the progression of diseases, including cancers (Wu et al., 2020). Emerging evidence indicates that ferroptosis participates in eradicating aggressive malignancies, thus offering opportunities for seeking a novel therapeutic biomarker in treating malignancies (Liang et al., 2019). Nowadays, emerging studies have identified diverse modulators and targets of ferroptosis for cancer research and treatment. For instance, acyl-CoA synthase 4 (ACSL4) is recognized as an essential regulator of ferroptosis, and inhibition of ACSL4 acts as a specific antiferroptotic rescue pathway (Kagan et al., 2017). Overexpression of metallothionein-1G (MT1G) mediates the occurrence of sorafenib resistance through repressing ferroptosis, indicating that targeting MT1G could ameliorate the anticancer activity of sorafenib (Sun et al., 2016a). However, the specific roles and underlying mechanisms of ferroptosis in LIHC have not been clearly unveiled.

The six-transmembrane epithelial antigen of prostate family member 3 (STEAP3) was initially discovered in murine myeloid M1 cells (Amson et al., 1996). STEAP3 was found to serve a key regulator in ferroptosis *via* mediating iron metabolism (Mou et al., 2019; Li et al., 2020). Recently, the vital roles of STEAP3 in cancers have been extensively studied. Over-expression of STEAP3 contributes to iron uptake and sustain iron storage in support of the proliferation of multiple cancer cells, including colorectal cancer (Barresi et al., 2016), pancreatic cancer (Yu et al., 2020), bladder cancer (Kim et al., 2016), etc. However, few studies have explored the roles of STEAP3 in prognosis as well as immune infiltration in LIHC.

In this study, we performed a detailed investigation to elucidate the detailed roles and potential mechanisms of STEAP3 in LIHC. Based on several public databases, the ferroptosis-related gene, STEAP3, was proved to impact the progression and prognosis of LIHC. Furthermore, silencing of STEAP3 was identified in the LIHC tissues and cells. The association between STEAP3 expression and clinical-pathological features was also investigated. Additionally, gene-set enrichment analysis (GSEA) was conducted to uncover its potential signaling pathways and biological functions. In addition, we analyzed the relationship between STEAP3 and tumor-infiltrating immune cells (TIICs) in LIHC. These results suggested the significant prognostic value of STEAP3 and an underlying target for immunotherapeutic approaches in LIHC.

## Materials and Methods

### Data Collection

We screened potential datasets from the Oncomine (Rhodes et al., 2007) according to the following inclusion criteria: (1) Cancer Type: LIHC, and (2) Analysis Type: Cancer vs. Normal Analysis. Three LIHC datasets from Oncomine were identified, such as GSE6764 (Wurmbach et al., 2007), GSE14520 (Wang et al., 2021) and GSE14323 (Mas et al., 2009). Then, these gene expression profiles were downloaded from Gene Expression Omnibus (GEO) database (Table 1; Clough and Barrett, 2016) and analyzed to screen the differential expressed genes between normal liver tissues and LIHC tissues. The cut-off value to screen the differential expression genes were as follows: *p*-value < 0.01 and | logFC| > 1. Next, to explore the role of ferroptosis-related genes in LIHC, Venn analysis was provided by Omicstudio^1^ to identify the co-differentially expressed genes (co-DEGs) among three GEO datasets and ferroptosis-related gene dataset (Liang et al., 2020).

**TABLE 1:** The primary characteristics of three GEO datasets on gene expression profiling *via* microarray.

**GEO^a^ datasets**	**Platform**	**Sample size**		**DEGs^b^**	**Co-DEGs**	**References**
		**Cancer**	**Normal**			
GSE6764	GPL570	35	10	830 up-regulated genes and 866 down-regulated genes	4 up-regulated genes and four down-regulated genes	Wurmbach et al., 2007
GSE14520	GLP3921	225	220	505 up-regulated genes and 590 down-regulated genes		Wang et al., 2021
GSE14323	GLP571	38	19	343 up-regulated genes and 258 down-regulated genes		Mas et al., 2009

*^*a*^GEO, Gene Expression Omnibus datasets.*

*^*b*^DEGs, differentially expressed genes.*

The TCGA, as a publicly open-access database, offers clinical and pathological information freely of 33 cancer types (McCain, 2006). Not only the gene expression profiles but also clinical-pathological information of LIHC patients were collected from TCGA platform within which 374 cases of LIHC and 50 adjacent-tumor samples were included.

### Bioinformatics Analyses

We applied Kaplan–Meier plotter to evaluate the prognostic value of co-DEGs in LIHC patients. The prognostic index includes the disease-specific survival (DSS), overall survival (OS) and progression-free survival (PFS). The Kaplan–Meier plotter, an online graphic tool, can explore the connection between gene expression and prognosis (Nagy et al., 2018). The expression levels of STEAP3 in LIHC tissues were analyzed *via* the following four databases. LIHC-related datasets, including GSE6764, GSE14520 and GSE14323 attained from GEO database, were utilized to analyze the STEAP3 expression levels among tumor and normal groups. Furthermore, the differential expression of STEAP3 was simultaneously validated using the TNMplot database and the Cancer Genome Atlas (TCGA) database. The TNMplot tool, comprising RNA-sequence data from TCGA and gene chip data from GEO, enables comparison of gene expression in cancerous, normal and metastatic tissues (Bartha and Győrffy, 2021). In addition, the protein expression of STEAP3 in liver tissue was explored through their immunohistochemical staining pictures obtained from the Human Protein Atlas (HPA) (Thul and Lindskog, 2018). And the relationship between STEAP3 expression and clinical characteristic parameters, such as T stage, M stage and histological grade, was also assessed in TCGA-LIHC dataset. What’s more, we used the pROC package (Robin et al., 2011) to assess the sensitivity and specificity of STEAP3 for the diagnostic significance of LIHC.

Next, LinkedOmics algorithm (Vasaikar et al., 2018) was exploited to analyze the 50 co-expressed genes associated with STEAP3. The results then were visually presented through volcano plots and heat maps. Furthermore, we also utilized LinkedOmics to perform the enrichment analysis of Gene Ontology biological process (GO-BP) and Kyoto Encyclopedia of Genes and Genomes (KEGG) pathways.

We performed the single-sample GSEA (ssGSEA) (Subramanian et al., 2005) to assess the relationship between the STEAP3 expression and 24 kinds of TIICs in LIHC. Additionally, we employed TISIDB (Ru et al., 2019) to repeat the immune infiltration cells related with STEAP3. Subsequently, immunohistochemical results gained from the HPA were used for validation of the association between STEAP3 expression and gene markers of immune infiltration cells. Finally, we also analyzed the associations between STEAP3 expression with immunomodulators and chemokines with TISIDB platform.

### Cell Cultures and Reagents

Liver hepatocellular carcinoma cell lines Huh7 and MHCC97H, and human immortalized hepatocyte cell line HHL-5 were cultured in a cell incubator including 5% CO_2_ at 37°C with Dulbecco’s modified Eagle’s medium (Gibco, United States) supplemented with 100 U/ml penicillin-streptomycin (Gibco, United States) and 10% fetal bovine serum (Gibco, United States). The STEAP3 plasmid was generated by cloning human STEAP3 cDNA into pcDNA3.1.

### RNA Isolation and Real-Time PCR

Total RNA was extracted utilizing Trizol reagent (Invitrogen, United States) and was reverse-transcribed to cDNA with the PrimeScriptTM strand cDNA synthesis kit (Takara, China). After then, to evaluate the expression level, real-time PCR was performed under the following thermal conditions: 95°C for 5 min, 40 amplification cycles of 95°C for 15 s and 56°C for 30 s. β-actin functioned as an internal control gene. β-actin primers were: forward 5′-CATGTACGTTGCTATCCAGGC-3′; reverse 5′-CTCCTTAATGTCACGCACGA-3′, and STEAP3 primers were: forward 5′-TGCAAACTCGCTCAACTGGAG-3′; reverse 5′-GAAGGTGGGAGGCAGGTAGAA-3′. The 2^–ΔΔ*Ct*^ method was applied for data analysis.

### Western Blot

In brief, the cell lysates were washed by phosphate buffered saline and prepared by lysis buffer (Thermo Scientific, United States). After 30 min of incubation on ice and centrifugation at 10,000 × *g* for 30 min at 4°C, protein concentrations were determined employing a BCA kit (Thermo Scientific, United States). Subsequently, proteins were separated using SDS-PAGE and transferred onto polyvinyl difluoride (PVDF) membrane (Millipore, United States). Membranes were blocked and incubated at 4°C overnight with primary antibodies as follows: anti-STEAP3 (1:250, Abcam, United States) and anti-β-actin (1:5,000, Santa Cruz, CA, United States), followed by secondary antibodies (1:10,000, Proteintech, United States). Finally, bands were detected using the Bio-Rad GelDoc XR + IMAGELAB system.

### Cell Counting Kit-8

Cell proliferation rates were analyzed using a cell counting kit-8 (CCK-8) assay. As previously reported (Chen et al., 2021), about 1,200 cancer cells were seeded in the 96-well plates. The CCK-8 test solution (B34304, Bimake, United States) was added into the culture solutions. After then, the cells were incubated in a cell incubator including 5% CO_2_ at 37°C for about 1 h. A spectrometer (PerkinElmer, United States) was used to measure the optical density at 450 nm in the 96-well plates.

### Colony Forming Assay

The Huh7 cells and MHCC97H cells were separately transfected with pcDNA3.1 or pcDNA-STEAP3 for around 48 h. Subsequently, a total of 1,000 cells were seeded in six-well plates and cultured for around 15 days. The visible colonies were counted through staining with crystal violet.

### Statistical Analysis

Statistical analysis was performed using the SPSS 19.0 software. A *p*-value of less than 0.05 was considered to be statistically significant. All experiments in this study were conducted at least three times with mean ± standard deviations (SD). Kaplan–Meier analysis was carried out to analyze survival rate for LIHC. The differential expression between LIHC and normal tissues were analyzed utilizing *t*-test. The relation between STEAP3 expression and clinicopathological variables was evaluated through the Fisher exact test, Chi-square test, and Wilcoxon rank sum test.

## Results

### The Differentially Expressed Genes Between Liver Hepatocellular Carcinoma and Normal Groups

We screened differential expression genes between LIHC and normal liver through analyzing the gene expression profiles from three GEO datasets, with the screening condition of *p* < 0.01 and | logFC| > 1. We identified 830 up-regulated genes and 866 down-regulated genes in GSE6764, 505 up-regulated genes and 590 down-regulated genes in GSE14520, and 343 up-regulated genes and 258 down-regulated genes in GSE14323, respectively (Supplementary Table 1).

**FIGURE 1:**
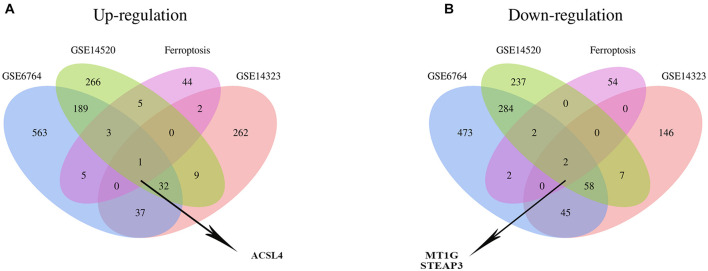
Identification of the co-DEG between three GEO datasets and the ferroptosis-related gene dataset. The number in every overlapping part meant the amount of differentially expressed genes. **(A)** One differentially expressed gene, namely ACSL4, was identified to be up-regulated in four datasets. **(B)** Two differentially expressed genes, including MT1G and STEAP3, were down-regulated in four datasets.

Recently, ferroptosis has been appreciated to participate in the progression of LIHC, which can ameliorate the resistance to antitumor drugs (Alu et al., 2020; Zhang W. et al., 2020). Thus, exploring the roles of ferroptosis in LIHC is becoming increasingly important. We, therefore, employed the Venn analysis to identify co-DEGs between the three GEO datasets and ferroptosis-related gene dataset. One up-regulated gene, ASCL4, and two down-regulated genes, MT1G and STEAP3, were preliminary identified, respectively (Figures 1A,B) and hypothesized to have potential roles in LIHC.

### STEAP3 Exhibits the Good Prognosis in Liver Hepatocellular Carcinoma Patients

**FIGURE 2:**
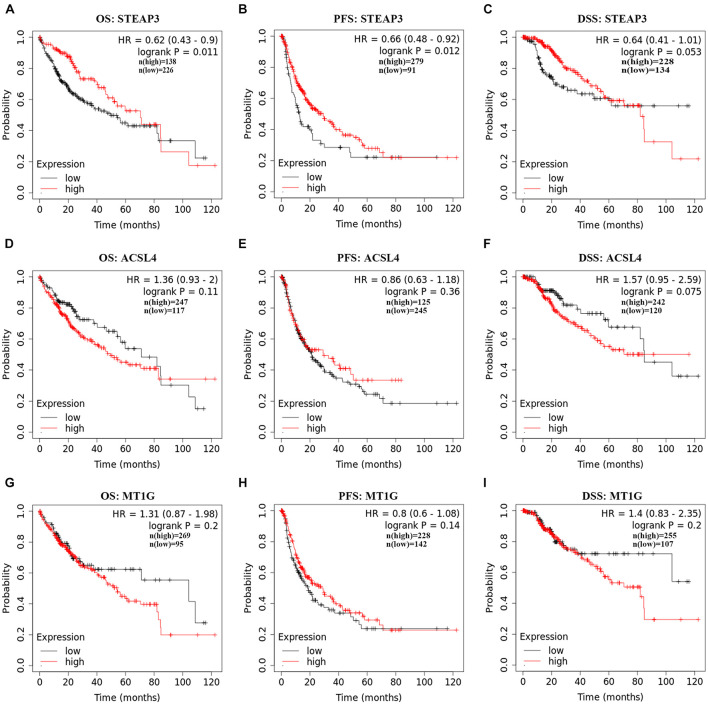
Prognostic values of ACSL4, MT1G, STEAP3 in LIHC. **(A–I)** The prognostic values of ACSL4, MT1G, STEAP3 in patients with LIHC using Kaplan–Meier plotter database. DSF, disease-specific survivor; OS, overall survival; PFS, progression-free survival.

The correlation between the expression level of ASCL4, STEAP3, and MT1G and their matching prognosis in LIHC patients were analyzed employing Kaplan–Meier plotter database. Of note, high STEAP3 expression was significantly associated with good OS (HR = 0.62, 95% CI = 0.43–0.9, *p* = 0.011) and PFS (HR = 0.66, 95% CI = 0.48–0.92, *p* = 0.012) (Figures 2A,B). Meanwhile, patients with high level of STEAP3 displayed slightly favorable DSS (HR = 0.64, 95% CI = 0.41–1.01, *p* = 0.053) (Figure 2C). However, there was no obvious relationship between the expression of ASCL4 or MT1G and prognosis in LIHC patients (*p* > 0.05) (Figures 2D–I). These results therefore clearly revealed that STEAP3 expression was significantly connected with great outcomes in LIHC and warrant a further study.

### Diminished STEAP3 Expressed in Liver Hepatocellular Carcinoma and Its Correlation With Clinicopathologic Features

**FIGURE 3:**
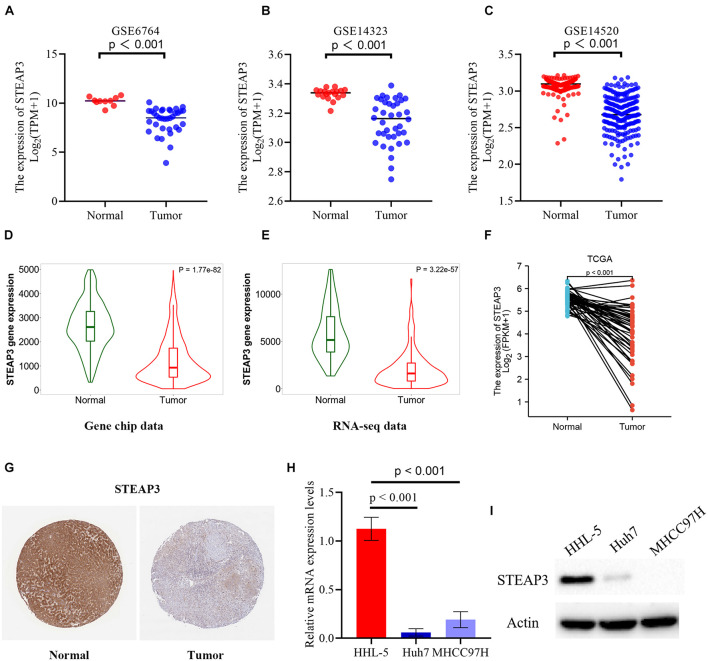
The expression level of STAEP3 in LIHC tissues and cells. **(A–C)** In comparison with normal liver tissues, STEAP3 expression was significantly diminished in LIHC tissues in three GEO datasets. **(D,E)** The expression level of STEAP3 was lower in LIHC compared with normal tissues from gene chip data and RNA-seq data of TNMplot database. **(F)** STEAP3 expression was dramatically restricted in LIHC compared to non-cancerous adjacent tissues from TCGA database. **(G)** Representative immunohistochemical image of STEAP3 in tumor tissues showed prominently diminished protein expression relative to normal tissues from HPA database. **(H,I)** STEAP3 levels were determined using PCR and western blot, respectively. β-actin was used for the loading control.

**FIGURE 4:**
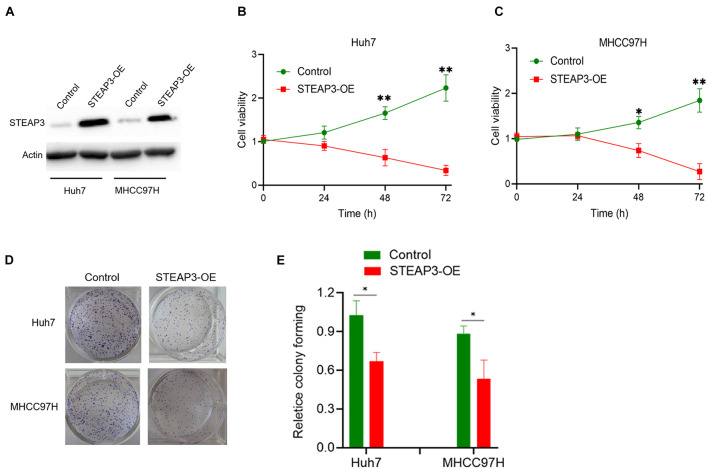
STEAP3 overexpression inhibited the cell proliferation. **(A)** LIHC cells Huh7 and MHCC97H were transfected with pcDNA3.1 or pcDNA-STEAP3, after which STEAP3 were evaluated by western blot. **(B,C)** CCK-8 assay indicated the effects of STEAP3 overexpression on the cell proliferation rate of Huh7 and MHCC97H cells. **(D,E)** STEAP3 overexpression inhibited the cell colony formation rate of Huh7 and MHCC97H cells. *p < 0.05, **p < 0.01.

From three independent LIHC studies of the GEO database, in comparison with non-cancerous tissues, the expression of STEAP3 was dramatically downregulated in tumor groups (*p* < 0.001) (Figures 3A–C). Then, analysis of STEAP3 expression with the TNMplot showed that STEAP3 mRNA expression was lower in cancer tissues from gene chip data (*p* = 1.77e-82) and RNA-seq data (*p* = 3.22e-57) (Figures 3D,E). What’s more, the differential expression of STEAP3 between LIHC and its corresponding non-cancer tissues can be confirmed by the data from TCGA (*p* < 0.001) (Figure 3F). Meanwhile, we assessed the protein expression levels of STEAP3 in LIHC patients *via* analyzing the immunohistochemical images from the HPA. As shown in Figure 3G, prominently decreased levels of STEAP3 was found in LIHC tissues. In addition, the downregulated STEAP3 was further identified in LIHC cells Huh7 and MHCC97H using PCR (*p* < 0.001) and western blot (Figures 3H,I). Moreover, STEAP3 was overexpressed in LIHC cells Huh7 and MHCC97H (Figure 4A). CCK-8 assay indicated that STEAP3 overexpression significantly inhibited the cell proliferation in Huh7 (*p* < 0.01) and MHCC97H cells (*p* < 0.01) (Figures 4B,C). Simultaneously, compared with the control group, STEAP3 overexpression significantly repressed the colony formation rate in Huh7 (*p* < 0.05) and MHCC97H cells (*p* < 0.05) (Figures 4D,E). These findings illustrated that STEAP3 expression was significantly attenuated in LIHC, revealing that STEAP3 may conduct its inhibitory function in the development of LIHC.

We then explored the connection between STEAP3 expression level and clinical information of LIHC patients, with the detailed clinical data attained from the TCGA database. As was shown in Table 2, STEAP3 expression was significantly related to histologic grade (*p* < 0.001), alpha fetal protein (AFP) (*p* < 0.001), as well as vascular invasion (*p* = 0.010). Furthermore, we analyzed the diagnostic value of STEAP3 to distinguish LIHC tissues from non-cancerous tissues utilizing the time-dependent receiver operation characteristic (ROC) curve. The area under the curve (AUC, AUC = 0.892, 95% CI = 0.861–0.923) indicated that STEAP3 had a great diagnostic value, and was hoped to be a potential biomarker for LIHC (Supplementary Figure 1).

**TABLE 2:** Association between clinical characteristic parameters and STEAP3 expression in LIHC patients from TCGA.

**Characteristics**	**Total (N)**	**Odds ratio (OR)**	***P* value**
T stage (T3 and T4 vs. T1 and T2)	371	0.777(0.484−1.244)	0.295
M stage (M1 vs. M0)	272	0.370(0.018−2.932)	0.392
Histologic grade (G3 and G4 vs. G1 and G2)	369	0.480(0.311−0.737)	< 0.001
Tumor status (With tumor vs. Tumor free)	355	0.794(0.521−1.209)	0.283
Gender (Male vs. Female)	374	1.025(0.664−1.582)	0.912
Race (White vs. Asian and Black or African American)	362	1.396(0.924−2.114)	0.114
Age (> 60 vs. ≤ = 60)	373	1.396(0.929−2.102)	0.109
Residual tumor (R1 and R2 vs. R0)	345	0.485(0.165−1.280)	0.158
Adjacent hepatic tissue inflammation (Mild and Severe vs. None)	237	1.015(0.606−1.699)	0.955
AFP (ng/ml) (> 400 vs. ≤ = 400)	280	0.348(0.192−0.616)	< 0.001
Albumin (g/dl) (≥ = 3.5 vs. < 3.5)	300	0.897(0.521−1.538)	0.694
Vascular invasion (Yes vs. No)	318	0.538(0.336−0.858)	0.010
Pathologic stage (Stage III and Stage IV vs. Stage I and Stage II)	350	0.697(0.429−1.128)	0.143

### STEAP3 Co-expression Network in Liver Hepatocellular Carcinoma

**FIGURE 5:**
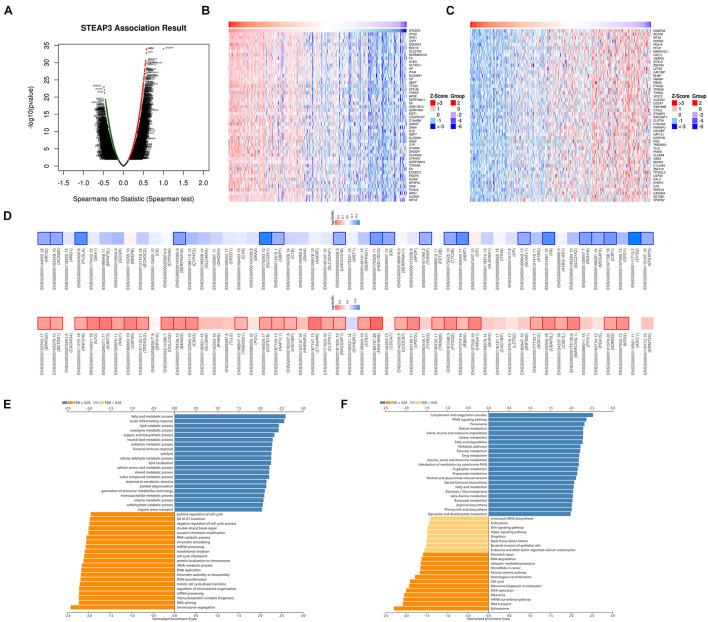
The co-expression genes of STEAP3 in LIHC. **(A)** The whole significantly correlated genes with STEAP3 identified with LinkedOmics in LIHC. **(B,C)** The top 50 genes displaying positive and negative association with STEAP3 in LIHC were showed in heatmaps respectively. The red represented positively connected genes and the blue meant negatively connected genes, respectively. **(D)** Survival heatmaps exhibited the top 50 genes bearing positive and negative correlation with STEAP3 in LIHC. **(E,F)** GO annotations as well as KEGG pathways of STEAP3 in LIHC cohort.

For investigating the biological meaning of STEAP3 in LIHC progression, the STEAP3 co-expression pattern in the TCGA-LIHC cohort was checked by deploying the function module of LinkedOmics. As shown in Figure 5A, it showed that 3,928 genes (red dots) positively related with STEAP3, and 5,836 genes (green dots) negatively associated (*p* < 0.05), respectively. The top 50 genes bearing positive and negative association with STEAP3 were shown in the heatmaps (Figures 5B,C and Supplementary Tables 2, 3). Notably, the top 50 positive genes exhibited high likelihood to become low-risk biomarkers in LIHC, of which 19/50 genes owned protected hazard ratio (HR). In contrast, the top 50 negative genes exhibited high likelihood to become high-risk biomarkers in LIHC. 32 of the top 50 negative genes had unfavorable HR (Figure 5D).

Gene Ontology term annotation showed that STEAP3-coexpressed genes joined mainly in fatty acid metabolic process, acute inflammatory response, lipid catabolic process, etc. (Figure 5E). KEGG pathway analysis suggested the enriched pathways were mainly involved in complement and coagulation cascades, PPAR signaling pathway, peroxisome, retinol metabolism, valine leucine and isoleucine degradation, carbon metabolism, and so on (Figure 5F).

### Correlation Between STEAP3 With Immune Infiltrations

**FIGURE 6:**
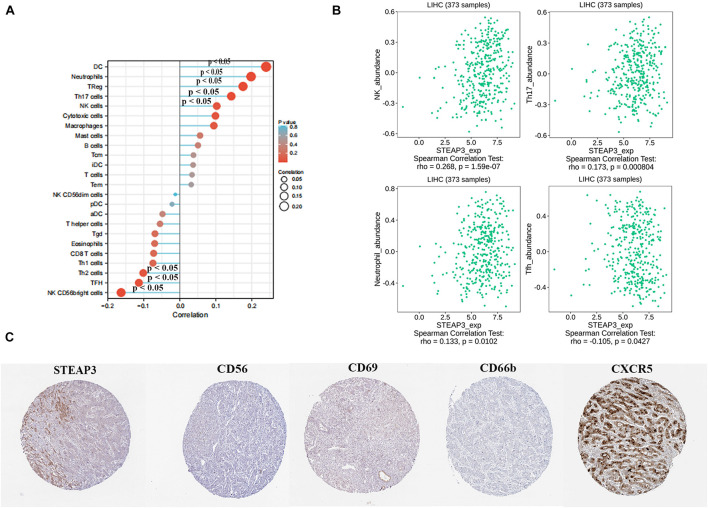
Correlation between STEAP3 expression with immune infiltration level in LIHC. **(A)** The association between STEAP3 expression level and 24 types of immune cells in TCGA database. Absolute values of Spearman r were measured by the size of round dots. **(B)** The scatterplot showed the association between STEAP3 and abundance of tumor-infiltrating lymphocytes (TILs), including NK cells, Th17 cells, neutrophils and Tfh cells. **(C)** Tumor infiltration of NK cells, Th17 cells, neutrophils and Tfh cells in liver cancer. STEAP3 staining, CD56 (NK marker), CD66b (neutrophils marker) were not detected, and CD69 (Th17 marker) showed low staining, while CXCR5 (Tfh marker) showed high staining.

Next, we analyzed whether STEAP3 expression can influence immune-associated functions in TCGA-LIHC cohort by ssGSEA with Spearman correlation. The results indicated that the expression of STEAP3 was positively associated with infiltration levels of natural killer (NK) cells, T helper type 17 (Th17) cells, and neutrophils (*p* < 0.05), and negatively related with T follicular helper (Tfh) cells (*p* < 0.05) (Figure 6A). Similarly, we reproduced immune infiltration profiles with TISIDB database and attained the consistent results (Figure 6B). We further explored the association between STEAP3 expression and gene markers of these tumor-infiltrating immune cells (NK, Th17, neutrophils and Tfh) by immunohistochemical images in LIHC tissues. The expression levels were quantitated by staining intensity, including not detected, low staining, medium staining and high staining. Intriguingly, in the LIHC tissues from a single provider, we found that the staining of STEAP3, CD56 (NK cell marker) and CD66b (neutrophils marker) were not detected, and the staining of CD69 (Th17 cell marker) staining was low. However, the staining of CXCR5 (Tfh cell marker) was high (Figure 6C). Taken together, the results revealed the positive correction between STEAP3 and the infiltration of NK cells, Th17 cells or neutrophils, and the negative correction between STEAP3 and the infiltration of Tfh cells.

Furthermore, to gain a better cognition of the association between STEAP3 and immune regulation, we explored the roles of STEAP3 in other immune signatures, such as immunostimulators, immunoinhibitors, chemokines and chemokine receptors. Supplementary Figure 2A displayed the association between STEAP3 expression and immunostimulators in LIHC patients. The immunostimulators showing the top four positively correlated molecules included TNFSF14 (Spearman *r* = 0.196, *p* = 0.000139), IL-6 (Spearman *r* = 0.19, *p* = 0.000232), CD40 (Spearman *r* = 0.158, *p* = 0.0022) and TNFRSF14 (Spearman *r* = 0.143, *p* = 0.00587) (Supplementary Figure 2B). Supplementary Figure 2C displayed the association between STEAP3 expression and immunoinhibitors. The immunoinhibitors that showed the top three negatively correlated molecules containing CTLA4 (Spearman *r* = −0.147, *p* = 0.0044), TGFB1 (Spearman *r* = −0.11, *p* = 0.033) and ADORA2A (Spearman *r* = −0.11, *p* = 0.0344) (Supplementary Figure 2D). As shown in Supplementary Figure 3A, we furthermore explored the correlation between STEAP3 expression and chemokines. The results exhibited the top four correlated molecules including CCL16 (Spearman *r* = 0.351, *p* = 4.09e-12), CXCL2 (Spearman *r* = 0.331, *p* = 7.39e-11), CCL14 (Spearman *r* = 0.262, *p* = 3.13e-07) and CCL23 (Spearman *r* = 0.248, *p* = 1.35e-06) (Supplementary Figure 3B). Additionally, Supplementary Figure 3C displayed the association between STEAP3 and chemokine receptors, of which CCR6 (Spearman *r* = −0.177, *p* = 0.000618), CCR10 (Spearman *r* = −0.148, *p* = 0.00413) and CXCR4 (Spearman *r* = −0.109, *p* = 0.0351) was obviously negatively correlated (Supplementary Figure 3D). Therefore, these findings indicated that aberrant STEAP3 has been proved to involve in modulating varieties of immune-associated signatures in LIHC.

## Discussion

Through this research, we aimed at exploring crucial and novel biomarkers that participated in the progression of ferroptosis in LIHC *via* several bioinformatics platforms which were summarized in Supplementary Table 4. Intriguingly, three genes including up-regulated ASCL4 and down-regulated MT1G and STEAP3 were identified by seeking co-DEGs in three GEO datasets and a ferroptosis-associated gene set. Unfortunately, diminished STEAP3 alone displayed a potential prognostic significance in LIHC patients. Moreover, ROC analysis indicated that STEAP3 could function as a diagnostic biomarker of LIHC. Therefore, our results revealed that STEAP3 was downregulated in LIHC and involved in the pathological progression of LIHC.

A highlight of this study is to predict the potential biological meaning by which STEAP3 regulates the progression of LIHC. Through LinkedOmics analysis, most genes co-expressed with STEAP3 in LIHC, either positively or negatively associated, also showed a dramatic relationship with LIHC patients’ prognosis. Additionally, these co-expressed genes were mainly involved in the regulation of metabolic microenvironment, which hinted that the metabolic regulation of tumor cells may be either direct or indirect through STEAP3 expression.

Ferroptosis, a newly discovered type of RCD, is featured by aberrant lipid peroxidation (Dixon et al., 2012). Recently, increasing researches have confirmed that inducing ferroptosis has emerged as a novel alternative to destruct cancer cells, especially drug-resistant malignancies such as LIHC (Su et al., 2020). Sorafenib, a Food and Drug Administration-Approved multikinase inhibitor applied for treating advanced LIHC (Wilhelm et al., 2008), was previously found to suppress system xc-mediated cystine import, and further trigger endoplasmic reticulum stress and ferroptosis (Dixon et al., 2014), which open up promising avenues for optimizing the application of sorafenib in LIHC (Louandre et al., 2013). More recently, haloperidol was identified to greatly promote sorafenib-induced ferroptosis *via* elevated Fe^2+^, glutathione (GSH), and lipid peroxides levels in LIHC cells and enhanced expression of glutathione peroxidase 4 (GPX4) and heme oxygenase-1 (HO-1), revealing patients who are diagnosed with LIHC and treated with sorafenib may gain benefit from the combination therapy of sorafenib and haloperidol (Bai et al., 2017). In addition to ferroptosis-inducer, various genes were found to function as regulators of ferroptosis in LIHC. Genes like Rb (Louandre et al., 2015), NRF2 (Sun et al., 2016b) and MT1G (Sun et al., 2016a) show the ability to inhibit sorafenib-induced ferroptosis, suggesting that suppressing these genes may ameliorate sorafenib resistance. Besides, other genes including CISD1 (Yuan et al., 2016) also serve as negative regulators of ferroptosis in LIHC, as well as polymorphism of TP53 genes which was also termed the S47 variant (Jennis et al., 2016). Apart from iron metabolism, likewise, lipid metabolism exerts a pivotal function in ferroptosis. For instance, low-density lipoprotein-docosahexaenoic acid nanoparticles (LDL-DHA) were demonstrated to induce ferroptosis *via* the occurrence of lipid peroxidation, depletion in GSH level and inaction of GPX4, thus substantially suppressing LIHC growth (Imai et al., 2017). The above-mentioned indicates that ferroptosis plays a pivotal role in developing and treating LIHC and in-depth exploring mechanisms might contribute to discovering novel therapeutic strategies. Nonetheless, the association between ferroptosis-associated genes and prognosis in LIHC patients has rarely been reported. In our work, we first investigated the prognostic significance of STEAP3, a ferroptosis-associated gene, in LIHC. We found that patients with highly-expressed STEAP3 displayed favorable prognosis.

STEAP3 is essential for transferrin (Tf)-mediated iron uptake, participating in the regulation of ferroptosis (Ohgami et al., 2005). STEAP3 may function as pro- and anti-cancer gene, which depends on the tissue type as well as the context (Grunewald et al., 2012). STEAP3 can be transcriptionally activated by p53 and thus enhance apoptosis as well as cell-cycle delay, revealing that STEAP3 might act as a tumor suppressor (Passer et al., 2003). Furthermore, ectopic expression of STEAP3 coupled with cisplatin exhibited additive effects on growth suppression and apoptosis in DU145 prostate carcinoma cells (Lu et al., 2009). In the opposite manner, loss of STEAP3 in glioma cells weaken the aggressive phenotypes accompanied by the inhibition of cell proliferation, invasion as well as sphere formation (Han et al., 2018). Similarly, in the present study, the ferroptosis-associated gene STEAP3 has been evidenced to be downregulated in LIHC tissues and cells.

There are accumulating evidences that tumor microenvironment as well as TIICs are currently topics of intense interest and have exhibited an essential role in cancer researches (Cabrita et al., 2020; Qiu et al., 2020). Cancer immunotherapy has remarkably revolutionized the strategies for cancer treatment by targeting the immune system, and then recognizing and disrupting cancer cells, therefore holds great promises for the prospect of deep, durable remission and potential cures of many patients (Cable et al., 2021). LIHC, as a lethal primary form of human liver cancer with an exceptionally poor prognosis, makes exploring novel therapeutic approaches against LIHC of paramount importance. Immunotherapies encompassing LIHC vaccines, immune-checkpoint inhibitors, adoptive cellular therapies as well as the application of oncolytic viruses for treating LIHC have emerged as promising alternatives for suppressing cancer progression, relapses and metastasis (Ghavimi et al., 2020). In this paper, assessment of the association between STEAP3 and the immune environment was presented. The finding implied that STEAP3 was positively associated with NK cells (marker: CD56), Th17 cells (marker: CD69) and neutrophils (CD66b) whereas inversely correlated with Tfh cells (marker: CXCR5). Furthermore, STEAP3 had the most significant relationship with immunostimulators (TNFSF14, IL-6, CD40 and TNFRSF14), immunoinhibitors (CTLA4, TGFB1 and ADORA2A), chemokines (CCL16, CXCL2, CCL14 and CCL23) and chemokine receptors (CCR6, CCR10 and CXCR4). Tfh cells, which are characterized by highly expressing CXCR5, could prime B cells to initiate antibody responses within extrafollicular and germinal center (Vinuesa et al., 2016). Increased number of Tfh cells contributes to immunosuppression and facilitates tumor development in LIHC and lower proportion of Tfh cells shows a better prognosis (Zhang et al., 2021). Furthermore, enhanced CXCR5 expression has been reported in LIHC with a higher fraction of CXCR5 + cells in poorly differentiated tumors relative to well-differentiated tumors, revealing that CXCR5 are associated with LIHC, potentiating possibilities for future immunotherapy (Duan et al., 2015). Genetic modification of NK cells projects a favorable light on developing effective immunotherapy against cancers through ameliorating their function and specificity (Xu et al., 2020). Human interleukin-15 gene-modified NK cells, a novel candidate for adoptive immunotherapy, have been shown to enhance the expressions of various cytolysis-related molecules such as interferon-γ (IFN-γ), Tumor necrosis factor-α (TNF-α), thus augmenting susceptibility of LIHC cells to NK-mediated cytolytic activity (Jiang et al., 2014). CCL14, a chemokine serving important roles in activating immune cells, was reported to be down-regulated in LIHC and exhibit a strong association with immune cells infiltration, hinting that it might be a potential biomarker determining the progression of LIHC (Gu et al., 2020). CTLA-4, an immune checkpoint molecule, can suppress T cells multiplication after recognized and differentiated by tumor-associated antigens (TAA) in many cancer including LIHC (Kudo, 2017). Moreover, CTLA-4 mediates immunosuppression inside LIHC tissues through the induction of regulatory T cells (Tregs) activity and the production of indoleamine 2,3-dioxygenase (IDO) and Interleukin-10 (IL-10) in dendritic cells (DCs) (Han et al., 2014). Nowadays, CTLA-4 antibodies have been identified in advanced LIHC patients with encouraging results in phase I-II-III studies (Donisi et al., 2020). Collectively, these findings reveal that STEAP3, which is linked with varieties of immune modules, plays an essential role in eliciting an immune response in LIHC microenvironments and may probably function as an immunotherapeutic target for LIHC.

## Conclusion

To sum up, this literature reported that downregulated STEAP3 displayed a significant association with favorable prognostic and diagnostic values in LIHC. Additionally, STEAP3 expression was remarkably correlated with the immune signatures, including immunostimulators, immunoinhibitors, chemokines and chemokine receptors. Therefore, we conclude that STEAP3, a ferroptosis-related gene, potentially plays a pivotal role in immune infiltration and serves as a favorable prognostic and diagnostic biomarker for LIHC patients.

## Data Availability Statement

The original contributions presented in the study are included in the article/Supplementary Material, further inquiries can be directed to the corresponding author/s.

## Author Contributions

QL and ZX: acquisition of data. JH and XC: analysis and interpretation of data. YC and BP: conception and design. QL, YC, and BP: data curation. QY: development of methodology. YY: writing the manuscript. All authors contributed to the article and approved the submitted version.

## Conflict of Interest

The authors declare that the research was conducted in the absence of any commercial or financial relationships that could be construed as a potential conflict of interest.

## Publisher’s Note

All claims expressed in this article are solely those of the authors and do not necessarily represent those of their affiliated organizations, or those of the publisher, the editors and the reviewers. Any product that may be evaluated in this article, or claim that may be made by its manufacturer, is not guaranteed or endorsed by the publisher.

## Footnotes

1
https://www.omicstudio.cn/index

## Supplementary Material

Supplementary Figure 1 ROC analysis of STEAP3 for LIHC patients. ROC curve showed the potential diagnostic value of STEAP3 as a biomarker in distinguishing LIHC tissue from healthy tissue. X-axis means false positive rate while Y-axis indicates true positive rate.

Supplementary Figure 2 Correlation of STEAP3 expression with immunomodulators in LIHC. **(A)** The association between STEAP3 expression and immunostimulators. **(B)** The top four immunostimulators bearing a positive correlation with STEAP3 expression. **(C)** The association between STEAP3 expression and immunoinhibitors. **(D)** The top three immunoinhibitors bearing a negative correlation with STEAP3 expression.

Supplementary Figure 3 Correlation of STEAP3 expression with chemokines or chemokine receptors in LIHC. **(A)** The connection between STEAP3 expression and chemokines. **(B)** The top four chemokines displaying relation with STEAP3 expression. **(C)** The connection between STEAP3 expression and receptors. **(D)** The top three receptors displaying relation with STEAP3 expression.
