# Supplementary figures and images for: Downregulated Ferroptosis-Related Gene STEAP3 as a Novel Diagnostic and Prognostic Target for Hepatocellular Carcinoma and Its Roles in Immune Regulation
Source: Front Cell Dev Biol. 2021 Nov 1;9:743046. doi: 10.3389/fcell.2021.743046 (PMC8591264; doi:10.3389/fcell.2021.743046)

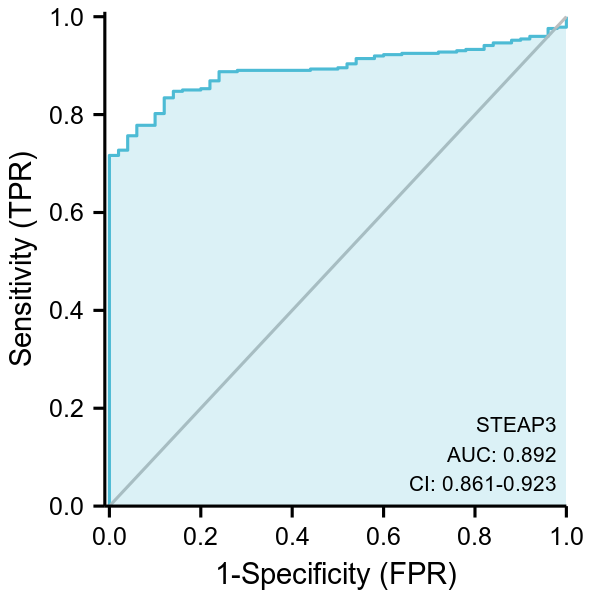

Supplement: Supplementary Figure 1 — ROC analysis of STEAP3 for LIHC patients. ROC curve showed the potential diagnostic value of STEAP3 as a biomarker in distinguishing LIHC tissue from healthy tissue. X-axis means false positive rate while Y-axis indicates true positive rate. [file Image_1.TIFF]

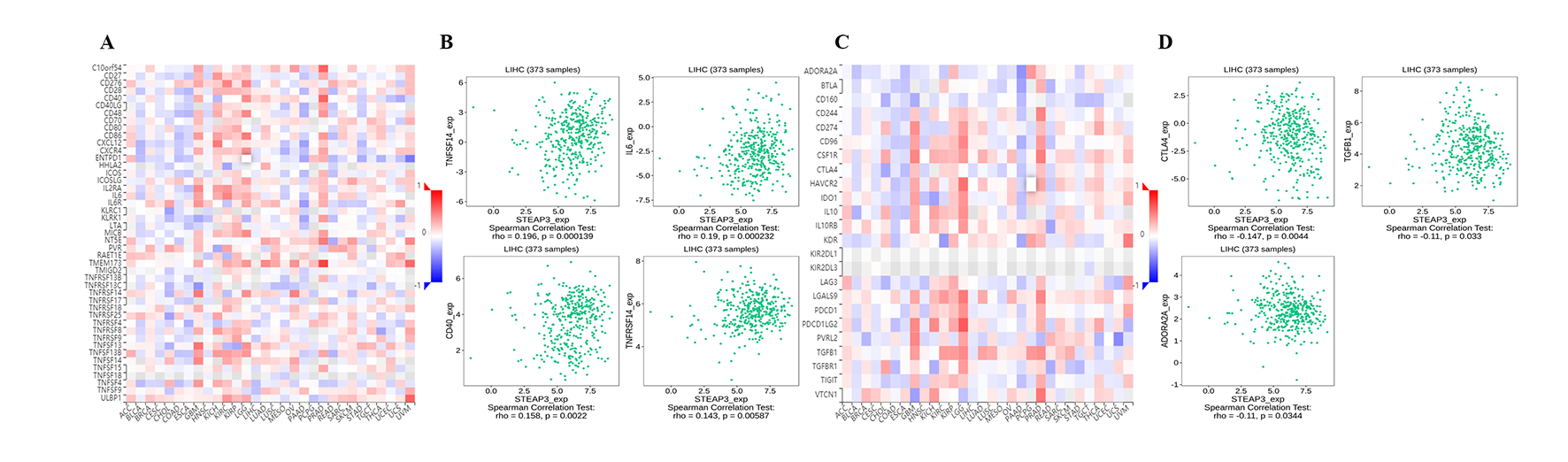

Supplement: Supplementary Figure 2 — Correlation of STEAP3 expression with immunomodulators in LIHC. (A) The association between STEAP3 expression and immunostimulators. (B) The top four immunostimulators bearing a positive correlation with STEAP3 expression. (C) The association between STEAP3 expression and immunoinhibitors. (D) The top three immunoinhibitors bearing a negative correlation with STEAP3 expression. [file Image_2.TIF]

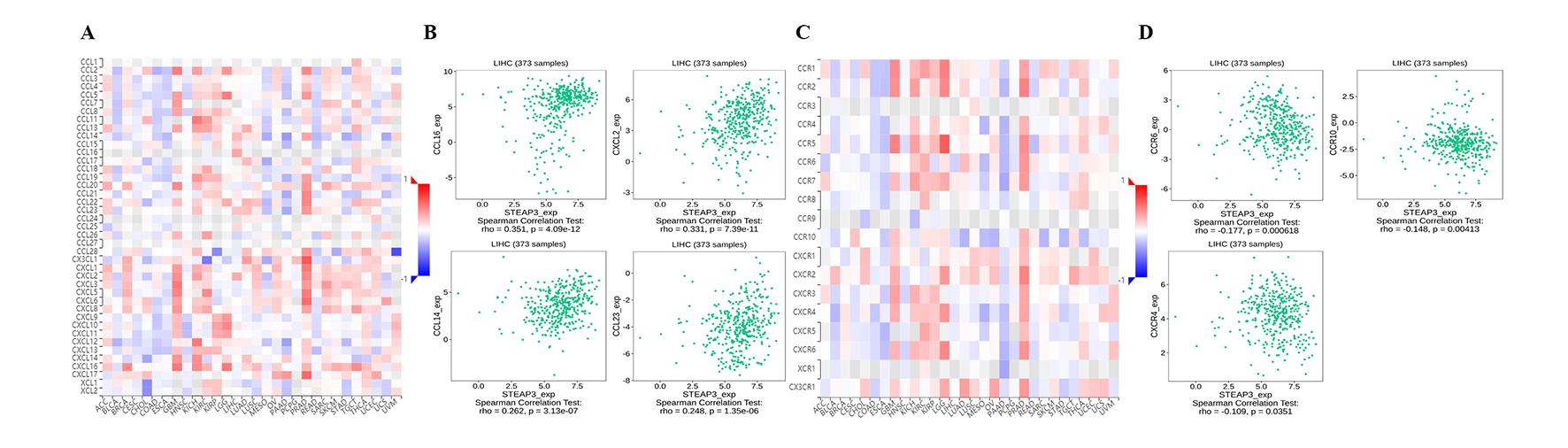

Supplement: Supplementary Figure 3 — Correlation of STEAP3 expression with chemokines or chemokine receptors in LIHC. (A) The connection between STEAP3 expression and chemokines. (B) The top four chemokines displaying relation with STEAP3 expression. (C) The connection between STEAP3 expression and receptors. (D) The top three receptors displaying relation with STEAP3 expression. [file Image_3.TIF]
