# Supplementary material for: Downregulated Ferroptosis-Related Gene STEAP3 as a Novel Diagnostic and Prognostic Target for Hepatocellular Carcinoma and Its Roles in Immune Regulation
Source: Front Cell Dev Biol. 2021 Nov 1;9:743046. doi: 10.3389/fcell.2021.743046 (PMC8591264; doi:10.3389/fcell.2021.743046)
Supplement: Supplementary file 5 [file Table_2.DOCX]

**Supplemental Table S2. The top 50 genes positively linked with STEAP3 in LIHC**

| STEAP3 | GLYATL1 | SERPINA11 | GBP7 | C9 |
| --- | --- | --- | --- | --- |
| GYS2 | HP | C6 | SLC22A1 | ECHDC2 |
| GPD1 | ITIH4 | HSD17B13 | SAA2 | REEP6 |
| C3P1 | ALDH8A1 | SERPING1 | C1R | GCGR |
| MOGAT2 | CP | MST1 | C7orf58 | MFAP3L |
| RDH16 | ABAT | LOC255167 | DHODH | SAA1 |
| SLC27A2 | TTC36 | C14orf68 | SLC46A3 | PLGLA |
| NCRNA00181 | FETUB | AADAT | CYP4F2 | ARG1 |
| F9 | THRSP | SAA4 | SERPINA10 | ACSM5 |
| A1BG | APOF | C1S | CYP2A6 | NR1I2 |
