# Supplementary material for: Downregulated Ferroptosis-Related Gene STEAP3 as a Novel Diagnostic and Prognostic Target for Hepatocellular Carcinoma and Its Roles in Immune Regulation
Source: Front Cell Dev Biol. 2021 Nov 1;9:743046. doi: 10.3389/fcell.2021.743046 (PMC8591264; doi:10.3389/fcell.2021.743046)
Supplement: Supplementary file 6 [file Table_3.DOC]

**Supplemental Table S3. The top 50 genes negatively linked with STEAP3 in LIHC**

| DNMT3A | ZNF530 | CCDC97 | NAP1L1 | ZNF416 |
| --- | --- | --- | --- | --- |
| ACCN2 | LZTS2 | CDCA7 | COPS7B | TPD52L2 |
| MTA3 | CACYBP | FAM189B | PGC | USP39 |
| NDRG3 | BLMH | CTSL2 | TMEM201 | RALY |
| PEA15 | HMGA1 | SYNGR1 | TLL2 | EHMT2 |
| PITX1 | RBMX | RACGAP1 | PI4KB | ILF2 |
| MARCKSL1 | PYGO2 | CLSTN1 | SLC6A8 | RNF216 |
| CSE1L | TRIM28 | C19orf48 | CBX3 | CACNG4 |
| UBAP2L | TYRO3 | HNRNPU | BEND3 | SETDB1 |
| SOX12 | VPS72 | HSF2BP | C11orf93 | SPATS2 |
