# Supplementary material for: Downregulated Ferroptosis-Related Gene STEAP3 as a Novel Diagnostic and Prognostic Target for Hepatocellular Carcinoma and Its Roles in Immune Regulation
Source: Front Cell Dev Biol. 2021 Nov 1;9:743046. doi: 10.3389/fcell.2021.743046 (PMC8591264; doi:10.3389/fcell.2021.743046)
Supplement: Supplementary file 7 [file Table_4.docx]

**Supplemental Table S4. Bioinformatic tools using for analyzing the roles of STEAP3 in LIHC.**

| Datebase | URL | Refs |
| --- | --- | --- |
| Oncomine | https://www.oncomine.org/resource/login.html | (21) |
| GEO | https://www.ncbi.nlm.nih.gov/gds/?term= | (25) |
| TCGA | https://portal. gdc.cancer.gov/ | (27) |
| Kaplan-Meier Plotter | http://kmplot.com/analysis/ | (28) |
| TNMplot | [http://www.tnmplot.com](http://www.tnmplot.com/) | (29) |
| HPA | http://www.proteinatlas.org/ | (30) |
| LinkedOmics | http://www.linkedomics.org/admin.php | (32) |
| TISIDB | http://cis.hku.hk/TISIDB/ | (34) |
